# Testing Shortened Versions of Smell Tests to Screen for Hyposmia in Parkinson's Disease

**DOI:** 10.1002/mdc3.12928

**Published:** 2020-03-21

**Authors:** Stephen D. Auger, Sofia Kanavou, Michael Lawton, Yoav Ben‐Shlomo, Michele T. Hu, Anette E. Schrag, Huw R. Morris, Donald G. Grosset, Alastair J. Noyce

**Affiliations:** ^1^ Preventive Neurology Unit, Wolfson Institute of Preventive Medicine, Barts and the London School of Medicine and Dentistry, Queen Mary University of London London United Kingdom; ^2^ Population Health Sciences, University of Bristol Bristol United Kingdom; ^3^ Oxford Parkinson's Disease Centre University of Oxford Oxford United Kingdom; ^4^ Nuffield Department of Clinical Neurosciences University of Oxford Oxford United Kingdom; ^5^ Department of Clinical and Movement Neuroscience UCL Institute of Neurology, University College London London United Kingdom; ^6^ Department of Neurology Institute of Neurological Sciences, Queen Elizabeth University Hospital Glasgow United Kingdom; ^7^ Reta Lila Weston Institute and Department of Clinical and Movement Neuroscience UCL Institute of Neurology, University College London London United Kingdom

**Keywords:** hyposmia, Parkinson's disease, UPSIT, smell tests, screening

## Abstract

**Background:**

Hyposmia is an early feature in neurodegenerative diseases, most notably Parkinson's disease (PD). Using abbreviated smell tests could provide a cost‐effective means for large‐scale hyposmia screening. It is unclear whether short smell tests can effectively detect hyposmia in patient populations.

**Objectives:**

To test the ability of short smell combinations to “prescreen” for probable hyposmia in people with PD and target administration of more extensive tests, such as the University of Pennsylvania Smell Identification Test.

**Methods:**

We assessed the screening performance of a short 4‐smell combination previously derived from use of the 40‐item University of Pennsylvania Smell Identification Test in healthy older people and its ability to detect hyposmia in a large cohort of PD patients.

**Results:**

The novel 4‐smell combination included menthol, clove, onion, and orange and had a sensitivity of 87.1% (95% confidence interval, 84.9%–89.2%) and specificity of 69.7% (63.3%–75.5%) for detecting hyposmia in patients with PD. A different (also novel) 4‐item combination developed using a data‐driven approach in PD patients only achieved 81.3% (78.2%–84.4%) sensitivity for equivalent specificity.

**Conclusions:**

A short 4‐smell combination derived from a healthy population demonstrated high sensitivity to detect those with hyposmia and PD.

Impaired olfaction (hyposmia) is an early feature of neurodegenerative diseases, most notably Parkinson's disease (PD).^1^, ^2^, ^3^, ^4^, ^5^, ^6^ The University of Pennsylvania Smell Identification Test (UPSIT), comprising 40 “scratch‐and‐sniff” microencapsulated odorant strips, is commonly used worldwide.^7^ We previously identified short smell combinations derived from the 40‐item UPSIT that could be cost‐effective for large‐scale hyposmia screening before targeted administration of the UPSIT.^8^ That work was conducted in the healthy general population recruited to the PREDICT‐PD cohort study and was compared with only a small sample of patients with PD. Here we tested the screening performance of several short smell combinations in the *Tracking Parkinson's* study that includes data from 1222 people with PD.^9^

## Methods

### Validating a Short Smell Test in People with PD

The performance of 5 smells (menthol, clove, onion, gingerbread, and orange) achieved a balance between brevity and high performance for identifying people with hyposmia against the full 40‐item UPSIT (sensitivity 94.1%) in the PREDICT‐PD pilot cohort's healthy participants (n = 891 for discovery and 191 for validation, as described previously^8^).

We assessed screening performance of a combination of 4 of these 5 smells, omitting gingerbread, in *Tracking Parkinson's* 1222 patients. This was because participants in PREDICT‐PD used the 40‐item US version of UPSIT, whereas *Tracking Parkinson's* cases completed the 40‐item UK version of UPSIT. These 2 UPSIT versions are broadly similar, but 8 of the 40 smells differ to tailor smells for recognizability in certain populations. We prioritized cross‐cultural smells (those appearing in both the US and UK UPSIT versions), which did not include gingerbread. A positive screen for hyposmia using this 4‐item combination was defined when 1 or more smells was identified incorrectly. Positive and negative screens for hyposmia were compared with hyposmia defined by performance on the full 40‐item test using the same age‐specific and gender‐specific thresholds described previously.^8^

### Deriving a Novel Set of Optimal Smells in the PD Cohort

We next ran a similar but abridged version of the full analysis we reported previously.^8^ We considered all possible combinations of 4, 5, and 6 smells from the full 40 UPSIT smells, testing multiple different score thresholds for defining hyposmia (ie, at least 1, 2, 3 etc. incorrectly identified smells to denote a positive hyposmia screen). These results were compared with the participants’ scores on the full 40‐item test in a “discovery” cohort comprising a randomly selected 90% of the participants from *Tracking Parkinson's* (n = 1100). The best‐performing combination of smells (defined by the highest sum of sensitivity and specificity) at each hyposmia threshold was tested in an independent “validation” cohort comprising the remaining 10% of participants (n = 122). We considered different proportions of data for discovery/validation (including 50% for each, 75%/25%, 80%/20%, and 95%/5%), but none achieved notably greater validation screening performance than 90% for discovery and 10% for validation. Screening performance of the best smell combinations is expressed as the values derived from the independent validation set.

### Additional Analyses

We next compared performance of the smell combinations used in the commercially available 4‐item “Pocket Smell Test” to our novel combinations using the same methods. There are 2 commercially available 4‐item “Pocket Smell Tests” (PST‐A and PST‐B); these are intended for use as a prescreen for identifying individuals who require a full UPSIT. The PST‐A includes chocolate, strawberry, smoke, and leather, whereas PST‐B includes grape, which is not included in the UK UPSIT and so was not further considered.

Finally, we assessed the ability of short smell combinations to distinguish people with PD (in *Tracking Parkinson's*) and those without (in PREDICT‐PD). For this analysis, positive and negative screens using the same criteria described previously were compared with whether the individual had a diagnosis of PD (rather than hyposmia or not).

## Results

### Validating a Short Smell Test in People with PD

Based on total UPSIT scores and age‐specific and sex‐specific thresholds, 80.9% of participants (988/1222) were classified as hyposmic in *Tracking Parkinson's*. At the time of olfactory testing, the PD patients had a mean disease duration of 1.89 years (range 0.36–4.50 years). Table 1 shows the screening performance of the menthol, clove, onion, and orange combination, where 1 or more incorrectly identified smell was used as a positive screen for hyposmia. A screen was considered negative if participants identified all 4 smells correctly. The corresponding screening performance values (and 95% confidence intervals) of the 4‐item combination were the following: sensitivity 87.1% (84.9%–89.2%), specificity 69.7% (63.3%–75.5%), positive predictive value (PPV) 92.3% (90.9%–93.7%), negative predictive value (NPV) 56.2% (51.7%–60.7%), positive likelihood ratio (LR+) 2.87 (2.36–3.49), and negative likelihood ratio (LR−) 0.18 (0.15–0.22).

**Table 1:** Screening performance of menthol, clove, onion, and orange using 1 or more incorrectly identified smell to define a positive screen, in 1222 people with recent‐onset Parkinson's disease

True Status	Positive Screen, n (%)	Negative Screen, n (%)	Total
Hyposmia	861 (87.1)	127 (12.9)	988
Normosmia	71 (30.3)	163 (69.7)	234
Total	932	290	1222

### Deriving a Novel Set of Optimal Smells in the PD Cohort

**Figure 1:**
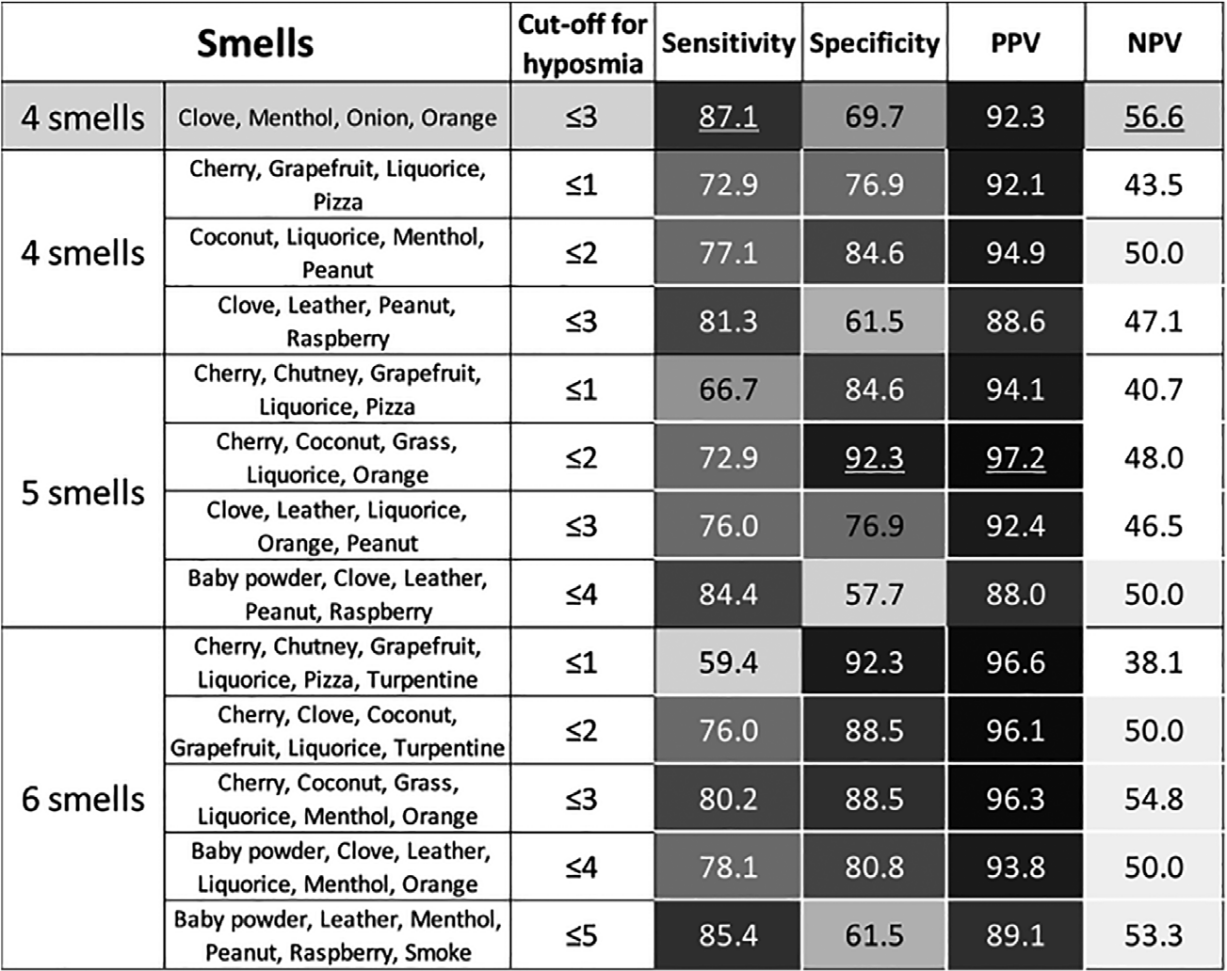
Screening performance in the validation cohort for each “winning” smell combination at the different cut‐offs used for defining hyposmia for subsets of 4 to 6 smells. The 4‐smell combination described in stage 1 is included at the top for comparison. Cell shading corresponds to the value of the number they contain. Values below 50 contain no shading, darker shading corresponds to higher values (in brackets of 5). The highest value in each category is underlined. PPV, positive predictive value; NPV, negative predictive value.

The optimum combinations of 4, 5, or 6 smells using different cut‐offs to define hyposmia (ie, ≤1, ≤2, ≤3 etc. correctly identified smells) as identified in a “discovery” cohort of participants is shown in Figure 1. None of these exceeded the sensitivity of the 4‐item combination used in stage 1 (Fig. 1), but some had higher PPV.

### Additional Analyses

For the commercially available PST‐A's combination of smells, the screening performance for detecting hyposmia on the UPSIT was the following: sensitivity 80.2% (77.5%–82.6%), specificity 72.6% (66.5%–78.3%), PPV 92.5% (90.9%–93.9%), NPV 46.4% (42.8%–50.1%), LR+ 2.93 (2.37–3.62), and LR− 0.27 (0.24–0.32). Accordingly, the novel 4‐smell combination from stage 1 possessed higher sensitivity for a similar specificity.

For detecting PD cases compared with controls, the screening performance of the menthol, clove, onion, and orange combination was the following: sensitivity 76.3% (73.8%–78.6%), specificity 69.0% (66.2%–71.8%), PPV 73.6% (71.7%–75.4%), NPV 72.0% (69.8%–74.22%), LR+ 2.46 (2.24–2.71), LR− 0.34 (0.31–0.38). As a comparison, the PST‐A smells had sensitivity 70.0% (67.4%–72.6%), specificity 60.1% (57.1%–63.0%), PPV 66.5% (64.6%–68.3%), NPV 64.0% (61.7%–66.2%), LR+ 1.75 (1.62–1.90), and LR− 0.50 (0.45–0.55); the full 40‐item UPSIT had sensitivity 80.9% (78.5%–83.0%), specificity 83.7% (81.4%–85.9%), PPV 84.9% (83.0%–86.6%), NPV 79.5% (77.5%–81.3%), LR+ 4.97 (4.33–5.71), and LR− 0.23 (0.20–0.26).

## Discussion

An abbreviated combination of 4 smells from the UPSIT (menthol, clove, onion, and orange) retained high sensitivity for identifying individuals with hyposmia in the context of PD. Previous work demonstrated that these 4 smells, as well as gingerbread, have high screening performance for detecting hyposmia in a general population and hinted toward similar screening performance in people with PD,^8^ an observation that is borne out in the current analyses. This combination of 4 smells also outperformed smells in the commercially available PST‐A in differentiating PD cases from controls (on account of high prevalence of hyposmia in PD). Although it performed less well when compared with the 40‐item UPSIT, these data suggest that abbreviated tests used as a screen in the prediagnostic phase of PD will not systematically miss a large proportion of those with PD‐related olfactory dysfunction.

Our proposed application for an abbreviated smell test would be as part of a 2‐step approach, whereby individuals complete an abbreviated test as a quick and cost‐effective prescreen to identify individuals who might be pertinent to consider for full‐smell testing. Maintaining high sensitivity would be most important for an abbreviated smell test used in this way to ensure that most people who require full testing are identified while allowing more cost‐effective, targeted administration of the full UPSIT. For example, sending a full UPSIT ($26.95 per test) to identify hyposmia in a cohort of 10,000 healthy older people would cost $269,500. Postage costs, based on UK pricing, would amount to $4 return for each participant ($40,000 total). Using this 4‐item prescreen ($3.95 per test), 31.0% in the PREDICT‐PD study screen positive and would also be sent a full UPSIT (total cost in tests $122,940). The 2‐step approach generates different postage costs as well. The lighter weight of a 4‐item test costs $2 return in the UK ($20,000 for 10,000 participants), with 31.0% incurring a second return postage cost of $4 for the full UPSIT ($12,400 for 10,000 participants). Hence, the total cost for nondiscriminative UPSIT administration versus using this 4‐item prescreen amounts to $309,500 versus $155,340, respectively. Given the 4‐item combination's 87.1% sensitivity for detecting hyposmia here, this halving of cost would come at the expense of missing approximately 10% to 15% of people in whom full UPSIT testing would have identified hyposmia.

The attempt to derive a novel “PD‐specific” abbreviated smell combination (stage 2) was notable for the much greater variability in smells featuring in winning combinations than was present using the PREDICT‐PD cohort's general population. This perhaps reflects greater variability and more erratic trends in olfactory dysfunction in PD cases than controls. Indeed, this is borne out in the fact that the 4 smells identified as performing best in PREDICT‐PD's general population outperformed the corresponding “winning” subset of 4 smells with hyposmia cutoff ≤3 derived from people with PD in every regard (sensitivity 87.1% vs. 81.3%, specificity 69.7% vs. 61.5%, PPV 92.3% vs. 88.6%, and NPV 56.2% vs. 40.7%, respectively). This adds further weight to the finding that the high‐performing smells identified in PREDICT‐PD's general population are a reliable marker for screening hyposmia.

Some limitations need to be noted. The high hyposmia prevalence (using UPSIT) in this cohort of people with PD (80.9%) is expected given the strong association between hyposmia and PD. In the PREDICT‐PD general population, hyposmia prevalence using the same criteria was 16.2%. This warrants attention as high prevalence drives higher PPV and lower NPV. Indeed, as is clear in Figure 1, PPV was consistently higher than NPV for every smell combination in the *Tracking Parkinson's* participants. Great care is necessary if attempting to extrapolate PPV/NPV to other populations given their high context dependency. There are likely to be other sociodemographic differences between PREDICT‐PD and *Tracking Parkinson's* participants in addition to disease status. These potential confounders could have influenced test performance, but the observation that the original 4 smells outperformed the data‐driven approach suggests this was limited. The analysis also extrapolated the performance of 4‐item smell tests from 4‐smell combinations taken within a larger 40‐item smell test. There is a chance that true performance with dedicated 4‐item tests may differ from the 4‐smell combinations identified in this work.

Our 4‐smell combination retains a degree of cross‐cultural relevance given that all 4 smells feature in both the UK and US versions of the UPSIT. However, testing here was only in individuals based in the United Kingdom, including a fairly restricted set of ethnicities. Further validation would be required including other ethnic minorities and in other countries to assess broader external validity. There also remain some unavoidable differences between the UK and US versions, most notably the use of different distractor options for some smells. In the analysis of screening performance for identifying PD cases versus controls, the cases (in *Tracking Parkinson's*) all completed the UK version of the UPSIT, whereas the controls (in PREDICT‐PD) completed the US version. This could have impaired performance of controls versus cases given that it was a UK population completing a US test version. However, this would likely have led to the underestimation rather than the overestimation of screening performance owing to a reduced difference between cases and controls.

Prospective follow‐up of these and similar cohorts will be able to provide additional information regarding possible clinical implications of having hyposmia in people already diagnosed with PD or whether disease duration is related to the incidence of hyposmia.

In conclusion, the abbreviated 4‐smell combination of menthol, clove, onion, and orange retains high sensitivity to detect those with hyposmia in the context of PD. This subset of smells has cross‐cultural relevance and outperformed attempts to derive a separate PD‐specific combination of smells for hyposmia screening.

## Author Roles

(1) Research Project: A. Conception, B. Organization, C. Execution; (2) Statistical Analysis: A. Design, B. Execution, C. Review and Critique; (3) Manuscript Preparation: A. Writing of the First Draft, B. Review and Critique.

S.D.A.: 1A, 1C, 2A, 2B, 2C, 3A, 3B

S.K.: 1C, 3B

M.L.: 1B, 3B

Y.B.‐S.: 1B, 3B

M.T.H.: 1B, 3B

A.E.S.: 1B, 3B

H.R.M.: 1B, 3B

D.G.G.: 1A, 1B, 3B

A.J.N.: 1A, 1B, 1C, 2A, 2C, 3B

## Disclosures

### Ethical Compliance Statement

Ethical approval was obtained from multicentre ethics committees and the relevant local research and development departments. Everyone participating in the studies provided written informed consent to have their data used for research upon enrolment. We confirm that we have read the Journal's position on issues involved in ethical publication and affirm that this work is consistent with those guidelines.

### Funding Sources and Conflict of Interest

There was no specific funding for this work. The Preventive Neurology Unit is funded by the Barts Charity. The PREDICT‐PD and *Tracking Parkinson's* studies are funded by Parkinson's UK. The authors declare that there are no conflicts of interest relevant to this work.

### Financial Disclosures for Previous 12 Months

S.D.A. receives salary from Barts Health National Health Service (NHS) Trust. S.K. has no financial disclosures. M.L. is employed on a Parkinson's UK grant. Y.B.‐S. received funding from National Institute of Health Research, Gatsby Foundation, Kidney Research UK, Medical Research Council (MRC), and Parkinson's UK. M.T.H. received honoraria from Roche and UCB Pharmaceuticals. A.E.S. is employed by University College London and NHS National Institute for Health Research UCL Biomedical Research Centre. She has received grants from the European Commission, Parkinson's UK, GE Healthcare, Economic and Social Research Council, International Parkinson's and Movement Disorders Society, University College London, National Institute of Health, and National Institute for Health Research University College London Hospitals (UCLH) Biomedical Research Centre; honoraria from Health Advances; advisory board fees from GE Healthcare, Roche, Biogen, and Bial; and royalties from Oxford University Press for *Rating Scales in PD*, University College London Business. H.R.M. is employed by University College London. In the past 12 months, he reports paid consultancy from Biogen, UCB, Abbvie, Denali, and Biohaven; lecture fees/honoraria from Biogen, UCB, C4X Discovery, GE‐Healthcare, Wellcome Trust, and Movement Disorders Society; research grants from Parkinson's UK, Cure Parkinson's Trust, PSP Association, CBD Solutions, Drake Foundation, and the Medical Research Council. H.R.M. is a coapplicant on a patent application related to the C9ORF72–Method for diagnosing a neurodegenerative disease (PCT/GB2012/052140). D.G.G. received honoraria from BIAL Pharma and Merz Pharm and grants from Parkinson's UK. A.J.N. receives salary from Barts Health NHS Trust and from the Barts Charity to support the Preventive Neurology Unit at the Wolfson Institute of Preventive Medicine, Queen Mary University of London (QMUL). A.J.N. has received grants from Parkinson's UK, the Leonard Wolfson Experimental Neurology Centre, the UCL Movement Disorders Centre, and the Virginia Kieley Benefaction. A.J.N. has received honoraria/consultancy fees from Britannia Pharmaceuticals, Global Kinetics Corporation, Profile Pharmaceuticals, Bial Pharmaceuticals, Biogen, and F. Hoffmann‐La Roche.
